# EFFECTIVENESS OF A COLLABORATIVE STEPPED-CARE MODEL FOR OLDER ADULTS WITH DEPRESSION

**DOI:** 10.1093/geroni/igz038.1892

**Published:** 2019-11-08

**Authors:** Terry Lum, Gloria Wong, Tianyin Liu, Shiyu Lu, Dara Leung, Lesley Sze, Joyce Sing, Weiwei Kwok

**Affiliations:** 1 Department of Social Work and Social Administration, PokFuLam, Hong Kong; 2 The University of Hong Kong, Pofulam, Hong Kong; 3 The University of Hong Kong, Hong Kong, Hong Kong

## Abstract

Background: Depression is common among older adults and creates a substantial burden on individuals, caregivers, and healthcare system. This paper presents an innovative collaborative stepped care intervention that promotes the coordination between elderly center and community mental health center to provide nonpharmacological intervention to elders with mild to moderate level of depression. Methods: The stepped care model were implemented in four districts in Hong Kong between September 2017 and February 2019. In each district, one community mental health center and one elderly center worked together to implement this stepped care model. A quasi-experimental design was used to study the effectiveness of this intervention. Findings: A total of 853 older adults completed the intervention and additional 500 elders were recruited as control. The average age of intervention participants was 76.3 years. Their levels of depression and anxiety were measured by Patient Health Questionnaire (PHQ-9) and Generalized Anxiety Disorder 7-item (GAD-7) respectively. The average intervention lasted for 10 months. Their average PhQ9 score reduced from 7.2 before intervention to 2.7 after intervention (t= 34.7, p < .001). Their level of anxiety was lowered from 4.9 to 2.0 (t=16.9, p < .001). The different between the intervention and control groups were statistically significant. Conclusion: The stepped care model was effective in reducing the levels of depression and anxiety among Chinese older people. This paper will give detailed information about the stepped care model and its implementation.

